# Warming deferentially altered multidimensional soil legacy induced by past land use history

**DOI:** 10.1038/s41598-018-19912-y

**Published:** 2018-01-24

**Authors:** Weiling Dong, Alin Song, Xueduan Liu, Bing Yu, Boren Wang, Yuqiu Lu, Yanling Li, Huaqun Yin, Jianwei Li, Fenliang Fan

**Affiliations:** 1 Key Laboratory of Biometallurgy of Ministry of Education, School of Minerals Processing and Bioengineering, Central South University, Changsha, 410083 China; 2 Key Laboratory of Plant Nutrition and Fertilizer, Ministry of Agriculture, Institute of Agricultural Resources and Regional Planning, Chinese Academy of Agricultural Sciences, Beijing, 100081 China; 3 National Engineering Laboratory for Improving Quality of Arable Land, Institute of Agricultural Resources and Regional Planning, Chinese Academy of Agricultural Sciences, Beijing, 100081 China; 4 Department of Agricultural and Environmental Sciences, Tennessee State University, Nashville, TN 37209 USA

## Abstract

The legacy effects of previous land use and climate history may affect current soil function. However, the manner in which these legacy effects of land use are modulated by the subsequent climate remains unclear. For this reason, we investigated how the legacies of soil multiple functions left by conversion of grassland to agricultural management were mediated by climate warming with a reciprocal transplant approach. The overall legacy was further separated into the contributions by changes in the abiotic properties of the soil (abiotic process) and microbial community (biotic process). We here hypothesized that warming may mediate the legacy effects of previous land use, mainly by changing biotic processes. Results indicated that warming significantly influenced the total legacies of soil respiration and three exoenzyme activities representing recalcitrant carbon, nitrogen, and phosphorus cycling, but did not affect the total legacy of β-1,4-glucosidase activity, which is involved in labile carbon cycling. The relative contributions of abiotic and biotic processes to the warming effects on the total legacy depended on the type of soil function. The effects of warming on land use change legacies were derived from altered bacterial community structure. The results of the present study suggest that climate conditions could interact with land use legacy to determine the ecosystem functions in a process-specific way.

## Introduction

Multifunction implemented by soil microbiome is essential to support the ecosystem services relevant to humanity, such as nutrients transformation, water cleaning, carbon sequestration^1,2^. A plethora of studies have documented that human activity significantly influences the soil biodiversity and function^1^. As the economy and environment change, some factors that originally affected the soil system cease to work or can be changed by other factors. For example, plough is abandoned to reduce soil erosion in slope farmland^3^ and monoculture is replaced by rotation to increase yield and nutrient use efficiency^4^. Some researchers have shown that the effect of the previous management by humans may persist, a phenomenon defined as a legacy effect^5^. There is a growing realization that legacy effects should be incorporated into current models to improve the predictability of soil functioning^6,7^.

The soil ecosystem is influenced by many factors besides previous and current human disturbances, including climate conditions. Seasonal temperature variation exceeds 10 °C in most terrestrial biomes. Soil moisture produced by the precipitation can differ greatly across wet and dry seasons during the yearly cycle. In addition, the temperature and precipitation would be further shifted to different extents in different regions when global climate change^8^. These factors could change numerous soil chemical and biochemical reactions and shape soil microbial community, consequently changing the overall soil function^9–11^. Studies have shown that climate conditions themselves can leave legacy effects on soil function^12^. However, it is unclear how the climate conditions interact with the legacy effect induced by changes in land use on soil function^13^.

Previous studies of soil legacy can be categorized into three groups. First, only soil abiotic properties, such as pH, cation exchange, and organic carbon, are affected by previous management history^14,15^. Second, only legacy induced by shift in the microbial community was monitored. For example, results showed that soil microbes from the home range (Europe) of the invasive exotic plant *Centaurea maculosa* L. have stronger inhibitory effects on its growth than soil microbes from where the weed has invaded in North America^16^. Third, these studies tended to investigate the overall legacy effect rather than examining abiotic and biotic components separately^6,11,17^. In most of these studies, only limited soil functions were investigated. It is largely unknown how legacy effects of multiple soil functions are left by previous soil management. The relative contributions of abiotic and biotic processes to the whole legacy effect are largely unclear^18^. It is also not clear whether changes in climate condition affect the legacy effects of land use by altering relative contribution of abiotic and biotic processes and that of the microbial community.

In order to support its large population, China has undergone intensive agriculture for a long time. The agricultural intensification has had significant undesirable influences on the soil system in many regions, such as soil acidification and erosion^3,15^. Therefore, it is urgent that more sustainable management practices, such as fallow, conversion of farmland to grassland, and hedgerow intercropping, should be adopted in these regions^3,19^. However, the manner in which these previous management practices will leave a legacy effect on soil function, especially under different climate conditions, is largely unclear. In the present study, we hypothesized that temperature mediates the legacy effects of previous land use majorly by changing biotic processes. Toward this end, the potential legacy effects of multiple soil processes induced by agricultural management at two temperatures (15 and 25 °C) were explored with the reciprocal transplant approach in a hilly red soil of southern China, where the soil is subject to severe erosion^20^. The contributions of abiotic and biotic processes to the overall legacy effects were assessed, and soil microbial communities were investigated with next-generation sequencing.

## Results

### Soil function

**Figure 1:**
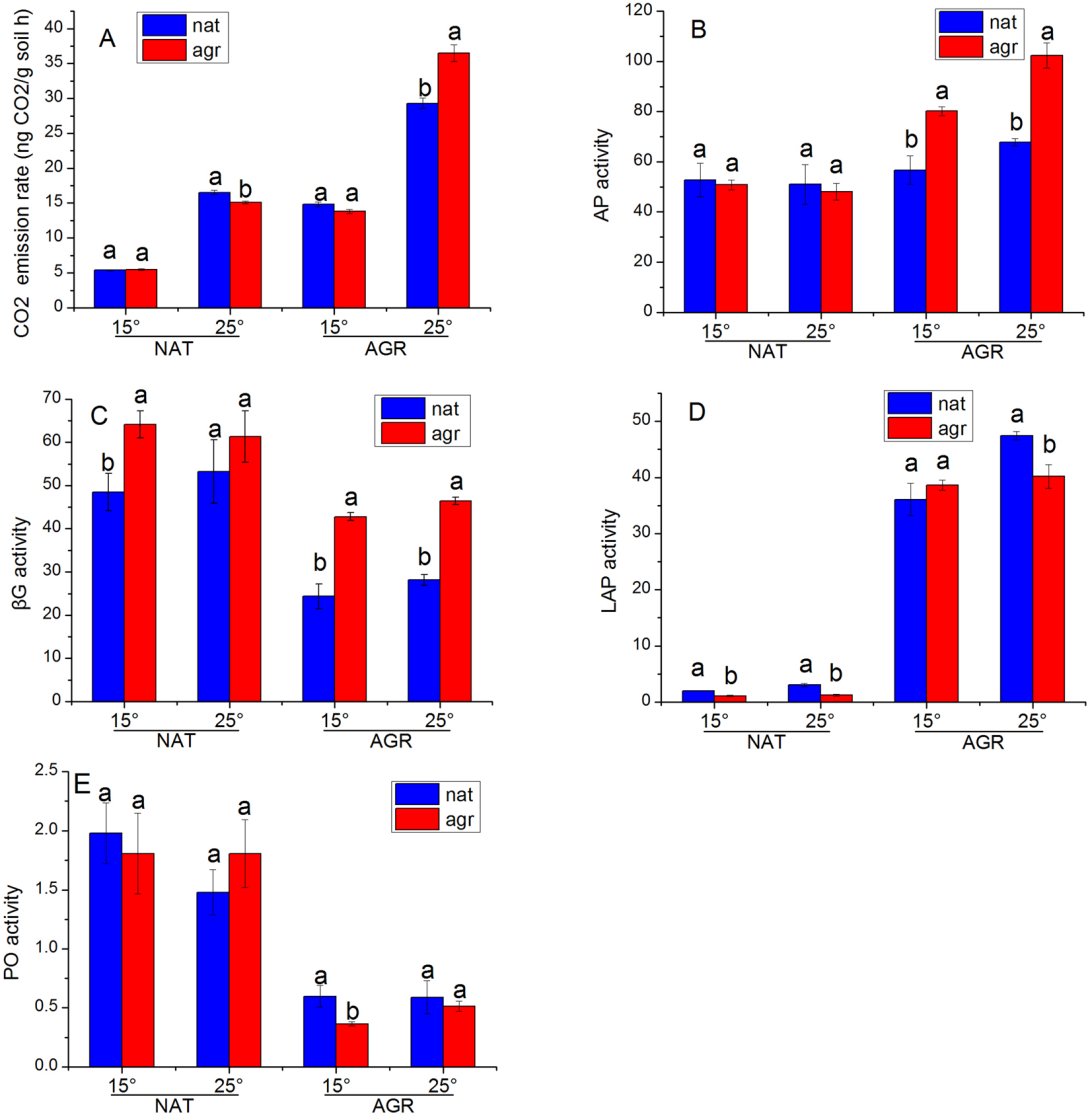
Soil microbial functions as affected by soil abiotic properties, microbiome and temperature. (**A**) Respiration; (**B**) acid phosphatase (AP); (**C**) β-1,4-glucosidase (βG); (**D**) leucine aminopeptidase (LAP) and (**E**) polyphenol oxidase (PO). Different letters indicate significant differences between microbiomes in each soil type-temperature combination. Error bars are standard error of the means with 5 replicates.

Changes in abiotic properties, microbiomes, and temperature, and their interaction effects on soil respiration rates were all significant (Fig. 1A). Shifts from natural grassland soil (NAT) to agricultural soil (AGR) and increases in temperature (from 15 to 25 °C) enhanced soil respiration. Shifts from natural soil microbiome (nat) to agricultural soil microbiome (agr) did not change soil respiration at 15 °C, but they did reduce respiration in NAT at 25 °C and increased respiration in AGR at 25 °C.

The overall effects of changing abiotic properties, microbiome, and temperature on acid phosphatase (AP) activity were all significant (Fig. 1B), but showing different patterns in AGR and NAT. In AGR, both shifts from nat to agr and increases in temperature increased AP activity. However, in NAT, the effects of shifts from nat to agr and increases in temperature on AP activity were not significant. Changing from NAT to AGR significantly reduced β-1,4-glucosidase (βG) activity, and changes from nat to agr increased βG activity. However, increases in temperature did not influence βG activity (Fig. 1C). Leucine aminopeptidase (LAP) activity was determined principally by soil abiotic properties (Fig. 1D). LAP activity in AGR was 8 fold higher than in NAT. In addition, the overall effect of temperature was positive. LAP activity at 15 °C and 25 °C in NAT and at 25 °C in AGR were higher with nat than with agr. The main effect of these changes on phenol oxide (PO) activity was only significant with respect to changes in soil abiotic properties (Fig. 1E).

### Legacy of soil function

**Figure 2:**
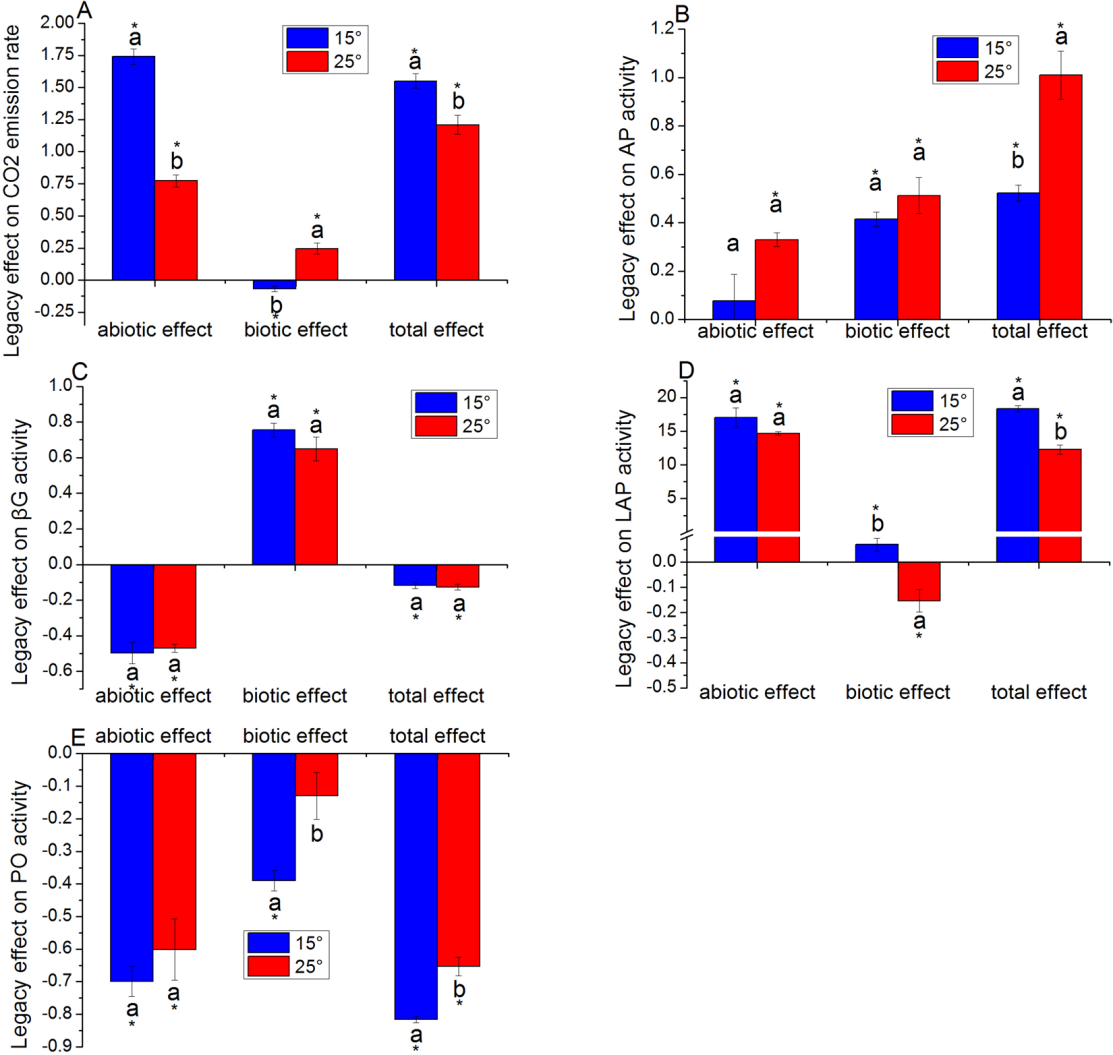
Abiotic and biotic process and total legacy effects on soil microbial functions at different temperatures. (**A**) Respiration; (**B**) acid phosphatase (AP); (**C**) β-1,4-glucosidase (βG); (**D**) leucine aminopeptidase (LAP) and (**E**) polyphenol oxidase (PO). Different letters indicate significant differences between temperatures for each legacy type. *Indicate a significant difference from zero. Error bars are standard error of the means with 5 replicates.

Agriculture had strong legacy effect on soil respiration and activities of the four exoenzymes (Fig. 2). The overall legacy effects on respiration (Fig. 2A), AP activity (Fig. 2B), and LAP activity (Fig. 2C) were positive, whereas the overall legacy effects on βG (Fig. 2D) and PO activity (Fig. 2E) were negative. The abiotic and biotic components of legacy were also different for each individual soil function. The abiotic and biotic components of legacy effects on βG activity had opposite effects. The total legacy effects of the soil respiration and the activity of the four exoenzymes, excepting AP, were majorly determined by abiotic processes associated with the legacy effect. However, the biotic processes associated with the legacy were also significant. In addition, changes in temperature significantly influenced total legacy of all the soil functions tested except for βG activity. Furthermore, changes in temperature significantly influenced biotic process of the legacy on respiration, LAP, and PO activities and abiotic processes of the legacy effects on respiration.

### Microbial abundance

**Figure 3:**
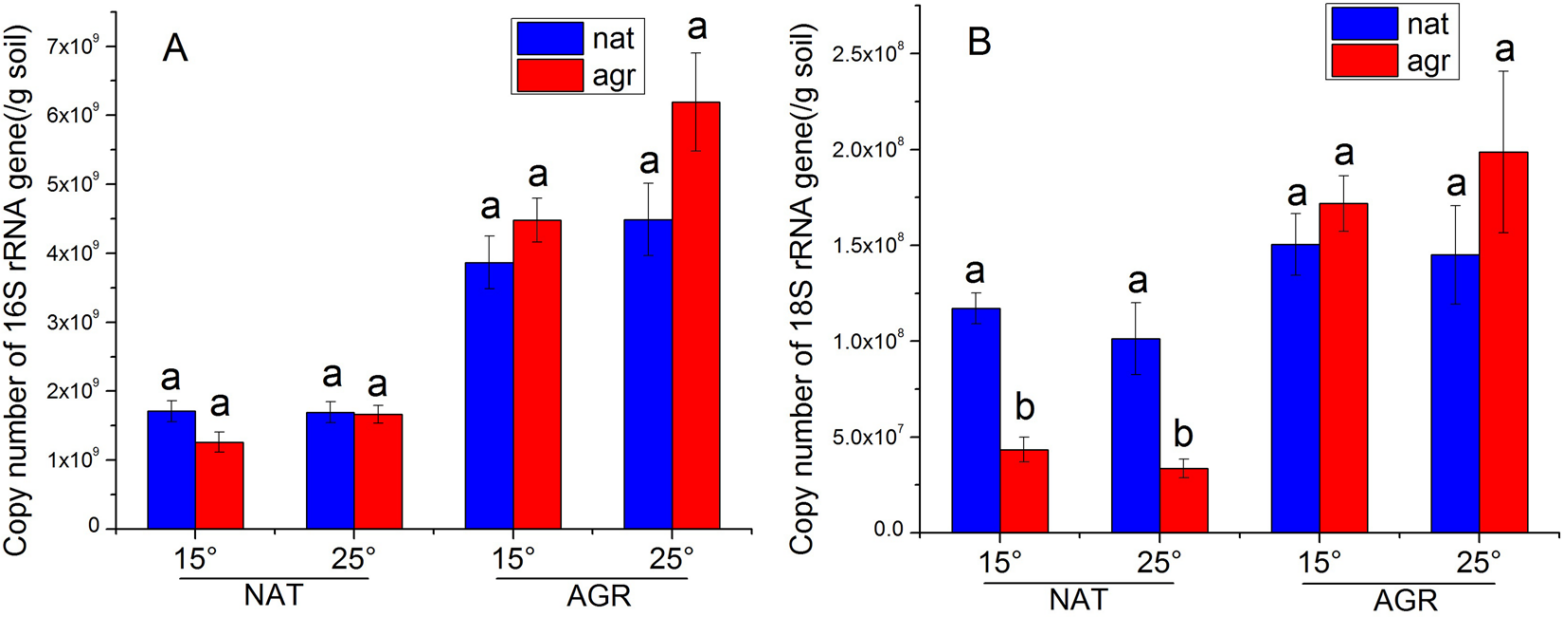
Microbial abundance as affected by soil abiotic properties, microbiome, and temperature. (**A**) Bacteria and (**B**) fungi. Different letters indicate significant differences between microbiomes in each soil type-temperature combination. Error bars are standard error of the means with 5 replicates.

Copy numbers of bacterial 16S rRNA gene in AGR was significantly higher than in NAT (Fig. 3A). Temperature and microbiome did not influence 16S rRNA gene abundance. Soil abiotic properties and microbiome significantly impacted copy numbers of fungal 18S rRNA gene, and their interactions were also significant (Fig. 3B). In NAT, copy numbers of fungal 18S rRNA gene with nat were significantly higher than with agr, but the effect in AGR was not significant.

**Figure 4:**
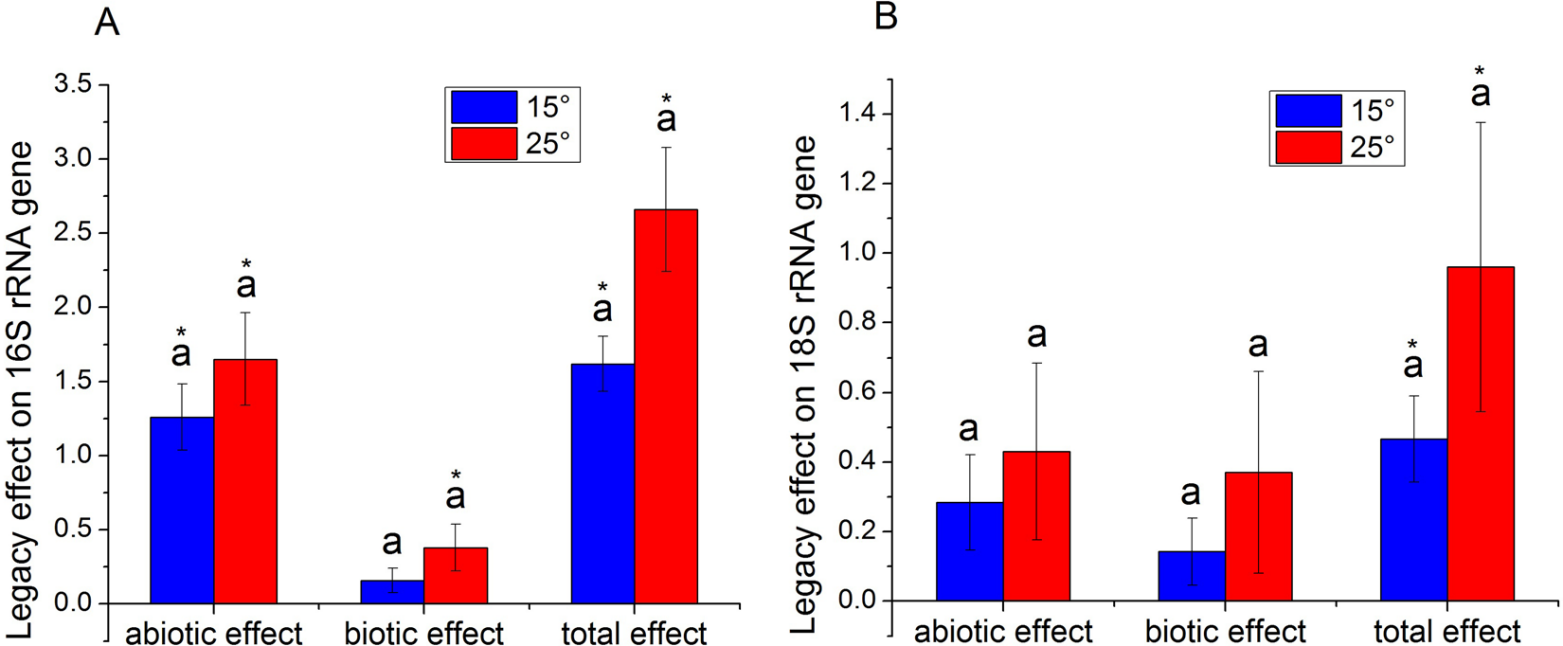
Abiotic and biotic processes and total legacy effects on microbial abundance at different temperatures. (**A**) Bacteria and (**B**) fungi. Different letters indicate significant differences between temperatures for each legacy type. *Indicate a significant difference from zero. Error bars are standard error of the means with 5 replicates.

The total legacy on copy number of bacterial 16S rRNA gene was positive (Fig. 4A). Abiotic process of the legacy on the copy number of bacterial 16S rRNA gene was similar to total legacy and higher than biotic process of the legacy. The total legacy on copy number of fungal 18S rRNA gene was also positive (Fig. 4B). Abiotic and biotic processes of the legacy on the copy number of fungal 18S rRNA gene were not significant.

### Microbial community structure and composition

**Figure 5:**
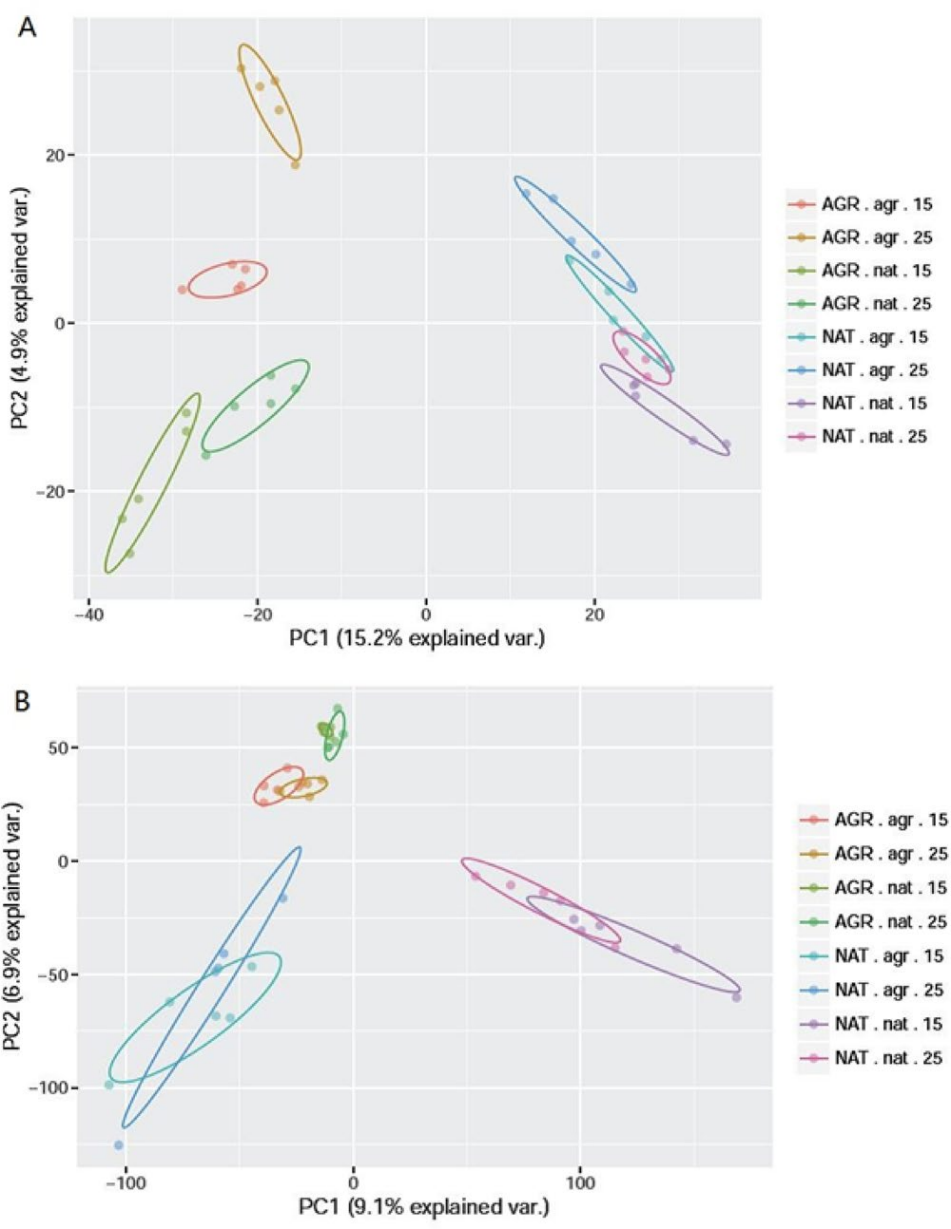
Principal component analysis of soil microbial community. (**A**) Bacteria and (**B**) fungi. AGR and NAT indicate agricultural and natural soils. agr and nat indicate microbiomes from AGR and NAT soils. 15 and 25 indicate 15 and 25 degrees Celsius (°C), respectively.

PCA analysis showed that soil bacterial community clustered according to soil abiotic properties and microbiome (Fig. 5A). The fungal community showed the same pattern as the bacterial community with a stronger temperature effect, but the effect of temperature on the bacterial community was relatively weak (Fig. 5B).

**Figure 6:**
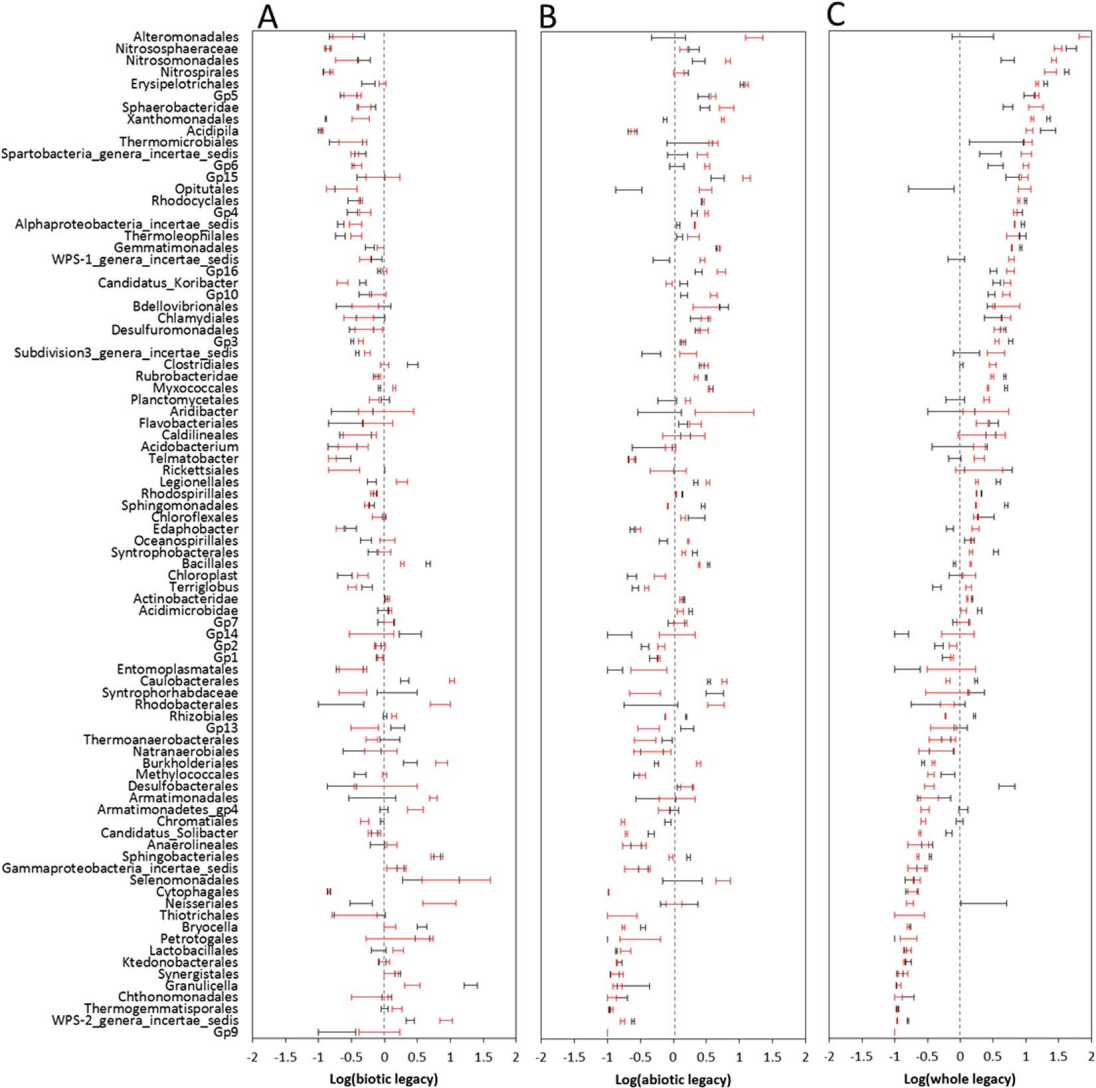
Abiotic and biotic processes and total legacy effects on relative abundance of each bacterial order at different temperatures. (**A**) Abiotic process; (**B**) biotic process; and (**C**) total legacy. Error bars are standard error of the means with 5 replicates. Black and red indicate 15 and 25 degrees Celsius (°C), respectively.

**Figure 7:**
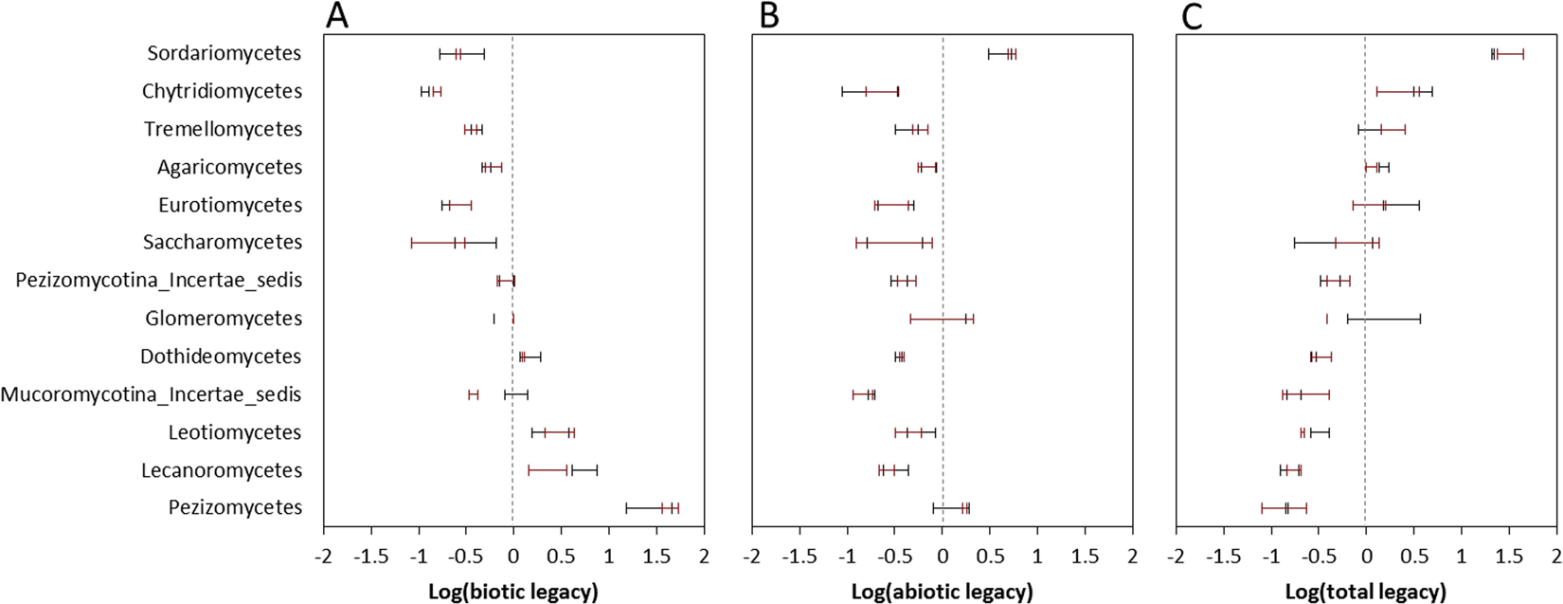
Abiotic and biotic processes and total legacy on relative abundance of each fungal order at different temperatures. (**A**) Abiotic process; (**B**) biotic process; and (**C**) total legacy. Error bars are standard error of the means with 5 replicates. Black and red indicate 15 and 25 degrees Celsius (°C), respectively.

We also analyzed the legacy effects on the relative abundance of each bacterial and fungal order (Fig. 6). The results showed that the total legacy effects on the bacterial taxa had a continuously positive to negative range. The abiotic and biotic processes of the legacy effects on the bacterial taxa were also apparent, but the total legacy effects were more similar to abiotic component of the legacy. The overall effects of temperature shift on the relative abundance of bacterial individual order were significant. Here, 48, 46, and 30 bacterial orders were significantly different at 15 °C compared with 25 °C for overall, abiotic, and biotic processes associated with the legacy effects, respectively. The total legacy effects on the relative abundance of fungal orders also showed a continuous positive to negative range, but the general patterns of abiotic and biotic processes of the legacy were different from those of the overall legacy effects (Fig. 7). In addition, the effect of temperature on the relative abundance of fungal orders was not significant.

## Discussion

In the present study, we found that agricultural management with conventional fertilization left a strong legacy in soil multifunction in this soil (Figs 1 and 2), which is consistent with the detection of land use and climate change legacy targeting to single soil function^7,10,21^. Strikingly, warming significantly influenced the total legacies of four of the five soil functions examined here. These changes were produced by converting grassland to agricultural use. Our results expand the earlier findings that legacy of land use can be influenced by change in additional environmental condition^7,18^. As far as we know, this is the first study to show that warming may influence legacy effects of changes in land use, demonstrating that future climate conditions could interact with previous human activity to mediate ecosystem functioning.

The influences of warming on the multidimensional legacy of changes in land use are complex (Fig. 2). First, the direction of the influence was found to vary. For instance, warming increased the total legacy of AP activity and decreased the total legacies of respiration, LAP, and PO activity. However, warming did not change the total legacy of βG activity. Second, the magnitudes of the warming effect on overall legacy effects were different for different soil processes. They ranged from 0% for βG activity to 89% for AP activity. Third, the warming effect on the total legacies of the same type of soil function could be different. Warming decreased the total legacy of the activity of PO, an exoenzyme degrading recalcitrant carbon, whereas it did not change the total legacy of the activity of βG, an exoenzyme that degrades labile carbon.

We then divided the overall legacy effects into abiotic and biotic processes. Results showed that the abiotic and biotic processes responded differently to warming. Warming only decreased the abiotic process of the legacy of respiration whereas had complex influences on biotic process of the legacies of the four soil functions. The responses of overall legacy to warming seem to be an additive effect of warming responses of the abiotic and biotic processes. For instance, an intermediate reduction in the total legacy of respiration was the result of sharp reduction in abiotic process with complimentary increase in biotic process in response to warming. An additive of slight increase in abiotic and biotic processes contributed to a substantial enhancement of the total legacy of AP activity. This partly supported our hypothesis that temperature mediates the legacy induced by previous land use by changing biotic processes.

The differences in shift direction and extent of the legacies of different soil functions suggest that the mechanism is complex. The outcome of the interplay between different unknown mechanisms in response to warming may reflect in the alteration of microbiome, so we characterized the community with qPCR and high-throughput sequencing. The methods could not directly bridge the microbial identity with function but did have functional implications because the microbial function was phylogenetically conserved^22,23^. The response of legacy to warming was principally reflected in the community structure of bacteria but not in the abundance of bacteria or community structure and abundance of fungi (Figs 4 and 5). In addition, the different responses of legacies of different soil functions were found to correspond to positive changes, negative changes, and negligible changes in abundances of some bacterial orders but not fungal orders (Figs 6 and 7). The results suggest that the response of legacy effects to warming was principally derived from an altered bacterial community structure.

The present study has some limitations. First, the soil samples were collected once to test the warming effect on the land use legacy. The results obtained from a single test do not necessarily indicate an effect at other time as soil microbiome is seasonally variable. Second, we set the treatment temperature to approximately the annual average mean temperature, which may not reflect the situation during cold winters and hot summers. Third, we applied temperature treatment after land use had continued for over 50 years. These results reflected how the land use legacies could be realized under different climate conditions. Actually the temperature may exert its influence at all times when the land was used in different ways. All efforts to overcome these limitations would further improve the understanding of how climate change could interact with land use type to determine ecosystem functioning in the future.

In summary, we investigated the temperature response of legacies of soil multiple functions after the conversion of grassland to farmland via the reciprocal transplant approach. We found that the direction and magnitude of legacies differed across individual soil functions. We found that warming had strong influence on the land use legacy, through changing both abiotic and biotic processes. Microbiologically, the responses of land use legacies to warming were derived from altered bacterial communities. The results of the present study suggest that other environmental conditions would interact with soil legacy of changes in land use to determine the ecosystem functions in a process-specific way. These new findings should be incorporated into the previous knowledge frame on soil legacy induced by changes in land use or climate change separately.

## Methods

### Site description and soil sampling

The agricultural farmland soil (AGR) and natural soil (NAT) used for this incubation experiment were collected from Qiyang County (26°45′12′′N, 111°52′32′′E), Hunan Province, southern China, after maize harvest at the end of August. The site has an average annual temperature and precipitation of 18 °C and 1250 mm, respectively. The soils are classified as ferralic cambisol and developed from quaternary red clay. The AGR and NAT samples were collected within 20 m of each other and were the same type of grassland about 50 years ago. AGR samples were collected from the conventional fertilization treatment in the long-term fertilization experiment of Chinese Academy of Agricultural Sciences which was previously described in detail^24^. Since 1990, the nitrogen application rate of the treatment was 300 kg N hm^−2^, with 70% from urea and 30% from pig manure N. The application rates of P_2_O_5_ and K_2_O fertilizers are 120 and 120 kg ha^−1^ year^−1^ in the form of calcium superphosphate and potassium chloride. The cropping system was a wheat-maize rotation cycle. NAT was collected from the grassland near the trial field used for the long-term fertilization experiment. The NAT had not ever been planted or supported a crop and its main vegetation was cogon grass (*Imperata cylindrica*). Soil was collected at several randomly chosen locations (5 cm diameter × 20 cm depth) and pooled to one composite sample to reduce heterogeneity. Fresh soil samples were first sieved at 2 mm and visible plant residues were removed by hand, adjusted to 60% water holding capacity, and stored in plastic bags. The soil properties of the two soils are listed in Table 1.

**Table 1:** Basic soil properties of natural and agricultural soils at Qiyang site, southern China.

Soil properties	Natural soil	Agricultural soil
pH (1:1 w/v water)	4.23	5.70
Soil organic C (g kg^−1^)	12.20	8.58
Total N (g kg^−1^)	2.10	1.07
Total P (g kg^−1^)	0.24	1.62
Available P (mg kg^−1^)	2.87	185.84
NH_4_^+^ concentrations (mg kg^−1^)	9.44	29.67
NO_3_^−^ concentrations (mg kg^−1^)	13.69	106.24

### Experimental design

The two sets of soil samples (NAT and AGR) were sterilized with 40 kGy gamma radiation. They were then inoculated with 7% of unsterilized AGR or NAT soil as a microbial inoculant (designated as agr and nat hereafter, respectively). In addition, 7% either AGR or NAT unsterilized soil was added with the unsterilized soil to minimize amendment of extra amounts of nutrients. After homogenization, the bags were closed with sterilized cotton stoppers to prevent microbial contamination while allowing for air exchange. They were then stored at room temperature (25 ± 2 °C) in the dark for 4 weeks to allow microbial regeneration. After that, soil equivalent to 30 g dry mass was sealed in a 125 ml serum bottles.

The experiment was carried out in a three-way random block design. The factors were as follows: temperature at two levels (defined as 15 °C and 25 °C, hereafter), soils at two levels (AGR and NAT), and soil microbiome at two levels (agr and nat). Each treatment was performed in 5 replicates. Then the bottles were incubated at 15 °C and 25 °C for 20 days in the dark. The temperature was set around the average annual temperature of the site (18 °C) where the soils were collected. Gas samples (10 ml) were taken from the headspace with a syringe at 2, 5, 10, 15, and 20 days after incubation. All bottles were ventilated for 5 min after gas was sampled, and the bottles were resealed. CO_2_ concentrations were measured with a gas chromatograph (HP7890A, Agilent Technologies, CA, US) as described by Fan *et al*.^25^. The bottles were destructively sampled at 20 days. Fresh soil samples were reserved for measurement of enzyme activity. The soil samples used for molecular analysis were stored at −80 °C.

### Measurements of soil exoenzyme activities

We measured four soil extracellular exoenzymes in the fresh soil. These enzymes are involved in carbon, nitrogen, and phosphorus cycling, including β-1,4-glucosidase (βG), polyphenol oxidase (PO), leucine aminopeptidase (LAP), and acid phosphatase (AP). We chose these four exoenzymes because they are the rate limiting factors in organic matter degradation^26–29^ and the three elements are the major nutrients that influence plant growth, soil fertility, greenhouse gas emissions, and many other ecosystem functions^24,30^. Enzymes targeting labile and recalcitrant carbons were selected because the decomposition of labile and recalcitrant carbons respond differently to global climate change^31^. These four enzymes have been widely used as parameters to represent soil functions in other studies^26,32–37^.

The exoenzymes were measured with a micro-plate exoenzyme assay as described by Saiya-Cork *et al*.^35^. Briefly, soil suspension was pipetted into a 96-well black plate with a multichannel pipette and continuously homogenized with a magnetic stirrer during the process. The sample well was given 50 μL 200 μmol·L^−1^ substrate linked with 4-methylumbelliferyl (4-MUB) and 200 μL soil suspension, the sample control well was given 50 μL deionized water and 200 μL soil suspension, the negative control well was given 50 μL substrate and 200 μL deionized water, the quench control well was given 50 μL standard materials (10 μmol L^−1^ 4-MUB) and 200 μL soil suspension, and the reference standard well was given 50 μL standard materials and 200 μL deionized water. The micro-plate was incubated at 25 °C for 4 h in the dark, and then given 10 μL of 0.5 M NaOH to terminate the reaction. Fluorescence was measured with 365 nm excitation and 450 nm emission filters using a microplate fluorometer.

### DNA extraction and real-time quantitative PCR (qPCR)

DNA extraction was performed using FastDNA spin kit for soil (MP Biomedicals) in accordance with the manufacturer’s instructions. The 0.5 g soil samples were prepared for DNA extraction to acquire enough DNA for subsequent testing. The extracted DNA was electrophoresed on 1.0% agarose gel and measured concentration using Nanodrop 2000 spectrophotometer (Thermo Scientific).

We performed qPCR assays to determine the abundance of DNA within the different samples. We used an ABI prism 7900 (Applied Biosystems) machine and SYBR green to quantify bacterial and fungal DNA obtained with primers that targeted regions of the 16S rRNA and 18S rRNA gene. For bacterial qPCR, each 15 μL reaction mixture contained the following: 7.5 μL of SYBR Premix Ex Taq (TaKaRa); 0.3 μL Rox (TaKaRa); 0.3 μL forward primer 357 F and 0.3 μL reverse primer 518 R; 4.6 μL pure H_2_O; and 2 μL DNA template. For fungal qPCR, each 15 μL reaction mixture contained the following: 7.5 μL of SYBR Premix Ex Taq (TaKaRa); 0.3 μL Rox (TaKaRa); 0.3 μL forward primer FF390, and 0.3 μL of reverse primer FR1. The primer pairs were tested and these PCR amplicons were used to generate a set of standards with known copy numbers of the target sequence. Standards were created by performing a 1:10 serial dilution to achieve a range from 10 to 10^8^ copies of the 16S rRNA or 18S rRNA gene. The coefficients of determination (r^2^) for our assays were 0.999 and 0.996 for the bacterial and fungal qPCR, respectively. The thermal cycle protocol was as follows: 95 °C at 3 min, followed by 40 cycles of 10 s at 95 °C, 30 s at 52 °C, and 45 s at 72 °C, with a 10 min final extension at 72 °C.

### Ion torrent sequencing

DNAs for microbial community determination were diluted to 2 ng μl^−1^. 16S RNA genes in the DNAs were amplified with primers 515 f and 806r (Peiffer *et al*. 2013) with a 12 bp barcode attached to the 5′ end of the reverse primer. Fungal internal transcribed spacer (ITS) regions were amplified with primers ITS3 and ITS4 with a unique 12 bp barcode attached to the 5′ end of primer ITS3. The 25 μl PCR mixture contained 2 μl template DNA, 2 μl dNTP, 0.5 μl forward and reverse primers, 0.25 μl rTaq (Takara), 2.5 μl 10 × rTaq buffer and 16.75 μl molecular biology grade water. All reactions were performed in triplicates using Biorad PCR machine (PTC 200, Biorad) with initial 94 °C denaturation for 4 min followed by 25 cycles of denaturing in 94 °C for 1 min, 55 °C annealing for 1 min, and 72 °C extension for 2 min with a final extension at 72 °C for 10 min. Equal mole of amplicons were pooled and gel purified with PCR amplicons purification kit (Tiangen Technologies). A library was constructed using an Ion Plus Fragment Library Kit and Ion PGM Template OT2 400 Kit and subsequently sequenced with an Ion PGM Sequencing 400 Kit and Ion 318TM Chip Kit v2 on an Ion Torrent PGM machine (Life Technologies).

Sequences with quality scores greater than 20 and without mismatches between the barcode and primer were processed further. The sequences were trimmed to 200 bp before clustering with UPARSE at a 97% similarity level^38^. Chimeras in the sequences were filtered with UCHIME^39^. The sequences analysis was performed using the USEARCH package^40^. Representative sequences were classified on RDP pipeline (http://pyro.cme.msu.edu/). An OTU table was rarefied to 2891 and 540 sequences per sample for 16S and ITS, respectively. The sequences were deposited in National Center for Biotechnology Information (NCBI) Sequence Read Archive (SRA) under accession number SRP074820.

### Statistical analysis

Total legacy was calculated as the relative change ratio of an individual function induced by the shifts in both soil abiotic properties and the microbiome as reported previously^12,13^. Abiotic processes associated with the legacy effects were calculated as the relative change ratio of an individual function induced by abiotic properties of soil samples inoculated with the same microbiome. Biotic process of the legacy was calculated as the relative change ratio of an individual function induced by inoculation of different microbiomes in the same sterilized soil. The calculation formulas were as follows:

$${\rm{Abiotic}}\,{\rm{effect}}=({\rm{AGR}}\,{\rm{with}}\,{\rm{nat}}-{\rm{NAT}}\,{\rm{with}}\,{\rm{nat}})/{\rm{NAT}}\,{\rm{with}}\,{\rm{nat}}$$

$${\rm{Biotic}}\,{\rm{effect}}=({\rm{AGR}}\,{\rm{with}}\,{\rm{agr}}-{\rm{AGR}}\,{\rm{with}}\,{\rm{nat}})/{\rm{AGR}}\,{\rm{with}}\,{\rm{nat}}$$

$${\rm{Total}}\,{\rm{effect}}=({\rm{AGR}}\,{\rm{with}}\,{\rm{agr}}-{\rm{NAT}}\,{\rm{with}}\,{\rm{nat}})/{\rm{NAT}}\,{\rm{with}}\,{\rm{nat}}$$

A three-way analysis of variance (ANOVA) was used to determine the effects of soil type, microbiome, and temperature on respiration, exoenzyme activity, and microbial community properties with SAS Windows version 9.1 (SAS Institute Inc., US). Principal component analysis (PCA) ordination of the relative abundances of OTUs detected in each sample was performed using a vegan package^41^ in an R statistics environment^42^. To examine the association of microbial community structure with function, the correlation between microbial community structure and soil functions was analyzed using Mantel test with the vegan package.
